# Diagnostic Performance of Corrected Carotid Flow Time for Assessing Fluid Responsiveness in Patients With Septic or Hypovolemic Shock: A Prospective Diagnostic Accuracy Study

**DOI:** 10.7759/cureus.117317

**Published:** 2026-10-01

**Authors:** Davis Cherian, Gaurav Jain, Ankit Agarwal, Barun Kumar, Bharat B Bhardwaj

**Affiliations:** 1 Anaesthesiology and Critical Care Medicine, All India Institute of Medical Sciences, Rishikesh, IND; 2 Cardiology, All India Institute of Medical Sciences, Rishikesh, IND; 3 Emergency Medicine, All India Institute of Medical Sciences, Rishikesh, IND

**Keywords:** corrected carotid flow time, diagnostic accuracy, echocardiography, fluid responsiveness, point of care ultrasound, septic shock

## Abstract

Background

Predicting fluid responsiveness in patients with hemodynamic instability remains challenging. Corrected carotid flow time (CFTc) is a non-invasive bedside Doppler measure proposed to identify fluid responsiveness, but its reported performance is inconsistent. We evaluated baseline CFTc as a predictor of fluid responsiveness and assessed whether the post-bolus percentage change in CFTc corresponded with the echocardiographic change in stroke volume.

Materials and methods

This prospective diagnostic accuracy study included 80 adult patients presenting with new-onset shock of presumed septic and/or hypovolemic origin. All received a clinically indicated first bolus of 10 mL/kg Ringer's lactate. Stroke volume (SV) measured by transthoracic echocardiography and CFTc by carotid Doppler were obtained at baseline (T1) and 20 minutes after the bolus (T2). Responders were defined by a ≥15% rise in SV. Receiver operating characteristic (ROC) curve analysis was performed to assess the diagnostic performance of CFTc and its percentage change.

Results

An infective focus was identified in 61 patients (76.2%), with no source documented in 19 patients (23.8%). Thirty-two patients (40.0%) were responders. A baseline CFTc cut-off of ≤324.03 ms predicted responders with 71.9% sensitivity and 47.9% specificity (AUROC 0.563, 95% CI 0.435-0.691; p = 0.343). The percentage change in CFTc showed poor discrimination for fluid responsiveness (AUROC 0.568, 95% CI 0.436-0.700; p = 0.305) and corresponded weakly with the percentage change in SV (Spearman rho = 0.141; p = 0.213). Non-responders experienced recurrent hypotension more often and required earlier initiation of vasopressors.

Conclusion

In adults with new-onset shock, baseline CFTc did not usefully discriminate echocardiography-defined fluid responders, and the post-fluid change in CFTc showed weak correspondence with the change in stroke volume. CFTc should not be relied on alone to guide fluid resuscitation in this population.

## Introduction

Effective fluid resuscitation is a cornerstone for effective management in patients with sepsis and other forms of shock, as emphasized by the Surviving Sepsis Campaign guidelines [1]. Clinicians must nonetheless weigh adequate volume replacement against the risk of fluid overload, a persistent diagnostic and therapeutic challenge. Inadequate resuscitation can result in inadequate tissue perfusion, whereas excessive fluid administration is increasingly linked to adverse outcomes, including acute kidney injury, interstitial edema, pulmonary edema, a prolonged need for mechanical ventilation, and increased mortality [1-4].

Critically, not every hypotensive patient benefits from additional fluid. Roughly 40-50% of hemodynamically unstable patients in the intensive care unit (ICU) fail to show a meaningful rise in cardiac output after fluid administration [3, 5]. Identifying in advance which patients are likely to benefit from fluid therapy is therefore essential, both to guide resuscitation and to avoid the harms of unnecessary volume loading.

Corrected carotid flow time (CFTc), a parameter measurable at the bedside with ultrasonography, has been proposed as a non-invasive tool for tracking real-time changes in volume status and predicting fluid responsiveness. CFTc can be obtained non-invasively at the bedside and may be technically less demanding than comprehensive echocardiographic hemodynamic assessment and may therefore be well suited to emergency and critical care settings [6-9]. Its reliability and diagnostic performance, however, may depend on operator experience, signal quality, vascular tone and the clinical population being evaluated.

Evidence to date is therefore mixed: several cohorts have reported good discriminatory performance for CFTc [6-9], while others studying more heterogeneous or acutely unstable populations have found its performance inconsistent [10,11].

Baseline CFTc and its post-bolus change address different clinical questions: the first can inform whether to give fluid, while the second can only describe what a bolus achieved. We therefore evaluated each separately against an echocardiographic reference standard. The primary objective was to determine the diagnostic performance of baseline CFTc for predicting fluid responsiveness. The secondary objective was to evaluate whether the post-bolus percentage change in CFTc corresponded with the echocardiography-derived change in stroke volume over the same interval. Lung ultrasound score, recurrent hypotension and vasopressor requirement within 24 hours were additional secondary outcomes.

## Materials and methods

This single-center, prospective diagnostic accuracy study was conducted at the All India Institute of Medical Sciences, Rishikesh, India, from March 2022 to June 2023, and was prospectively registered with the Clinical Trials Registry (CTRI/2022/03/040761). After approval by the institutional ethics committee and written informed consent, all patients aged 18-65 years with new-onset shock (mean arterial pressure [MAP] <65 mmHg) of presumed septic and/or hypovolemic origin who met protocol criteria were enrolled at their first presentation to emergency or ICU. All enrolled patients were spontaneously breathing at the time of study assessment. The study adhered to the principles of the 2013 Declaration of Helsinki.

Patients were excluded if they had a body mass index (BMI) >30 kg/m², cardiac arrhythmia, valvular heart disease, cardiomyopathy, carotid artery stenosis, chronic obstructive pulmonary disease (COPD), symmetrical anterior B-lines on lung ultrasound, cardiogenic or obstructive shock, vasopressor use at admission, or an inadequate transthoracic or carotid acoustic window. These criteria standardized acquisition and limited confounding of flow-time measurement by non-preload factors.

Fluid resuscitation protocol

All patients received standard resuscitation according to institutional guidelines. Initial resuscitation consisted of a clinically indicated 10 mL/kg bolus of intravenous (IV) Ringer's lactate given over 20 minutes, followed by repeat lung ultrasound and echocardiographic assessment. Patients with a ≥15% rise in SV 20 minutes after the initial bolus were classified as responders. This threshold was chosen because the least significant change in left ventricular outflow tract velocity time integral (LVOT VTI) measurable by a single operator after a 500 mL fluid bolus has been reported as 11% (range 5-18%), so a 15% threshold exceeds the expected measurement error [12]. Additional fluid boluses were given using the same protocol until one of the following occurred: MAP reached ≥65 mmHg, three boluses had been given, or new-onset anterior B-lines appeared on lung ultrasound. Persistent hypotension thereafter was managed with vasopressors or further fluids at the discretion of the treating physician. Patients were followed for 24 hours to identify recurrence of shock, defined as MAP <65 mmHg requiring a further fluid bolus or vasopressor after initial resolution.

The primary variables were CFTc and SV measured at baseline (T1) and 20 minutes following the first fluid bolus (T2). Secondary variables were the lung ultrasound score (LUS) and SV at T1, T2, T3 (post 2nd bolus) and T4 (post 3rd bolus), together with the incidence of recurrent hypotension and vasopressor requirement within 24 hours.

At baseline (T1), screening echocardiography and lung ultrasound were performed first, followed sequentially by LVOT VTI and Carotid Doppler. These assessments took place concurrently with initial resuscitation and consent discussion. After the bolus, LVOT VTI and CFTc were acquired first at T2, followed by lung ultrasound and vital signs. No changes were made to sedation, ventilation or position between T1 and T2.

Reference standard and index test

All echocardiographic assessments were performed by a single intensivist trained in critical care echocardiography consistent with American Society of Echocardiography recommendations during their own duty hours (convenience sampling) [13]. The same operator obtained pre- and post-fluid-bolus measurements in each patient to minimize measurement variability. Patients were positioned in the left lateral decubitus position where feasible. Images were acquired with a 5-11 MHz phased-array probe (Sonosite M-Turbo, FUJIFILM Sonosite, USA). The index test (carotid Doppler CFTc) and the reference standard (echocardiography-derived SV) were obtained during the same session by a single unblinded operator. Echocardiographic images were reviewed by a cardiologist who confirmed image quality and the accuracy of the stroke volume measurement. The cardiologist was blinded to time point, clinical status, CFTc values and responder classification.

Left ventricular outflow tract diameter and left ventricular outflow tract velocity time integral

Left ventricular outflow tract (LVOT) diameter was measured in the parasternal long-axis view, with the image optimized and zoomed to visualize the central axis of the LVOT and the aortic valve (AV) cusp insertion points (the annulus). Measurements were taken 3-10 mm from the AV plane in mid-systole. LVOT velocity time integral (VTI) was measured in the apical five-chamber view. A pulsed-wave Doppler sample was positioned approximately 5 mm proximal to the AV, at the center of the LVOT, and the spectral Doppler signal was traced to calculate the VTI; measurements were averaged over three consecutive cardiac cycles. SV was then derived from these measurements using the standard formula, SV = 0.785 × (LVOT diameter) ² × LVOT VTI [13].

Carotid Doppler assessment

With the patient supine and the neck in a neutral position, the right common carotid artery was scanned longitudinally with a high-frequency linear probe: the artery was first identified in short axis, then the probe was rotated 90° to obtain a long-axis view. Pulsed-wave Doppler was performed just proximal to the carotid bulb, with a 60° angle correction and a 2 mm gate size. Carotid flow time (CFT), the interval from the Doppler upstroke to the dicrotic notch, and cycle time, the interval between successive Doppler upstrokes, were recorded, and CFTc was derived using Bazett's formula (CFTc = CFT/√cycle time), with CFT in milliseconds and cycle time converted to seconds; this correction was chosen because it remains the heart-rate correction method most widely applied in carotid flow time studies, allowing direct comparison with previously published literature [6-9]. Measurements were taken over three consecutive cardiac cycles, and the mean value was used for analysis. Cycles with an indistinct dicrotic notch, marked respiratory variation, ectopy or a suboptimal Doppler envelope were excluded from the average and acquisition repeated.

Lung ultrasonography was performed using a 2-5 MHz phased-array probe. Each hemithorax was assessed across six regions - anterior, lateral, and posterior (superior and inferior zones) - and patterns were classified as N, normal aeration (A-lines); B1, moderate aeration loss (well-separated B-lines); B2, severe aeration loss (coalescent B-lines); or C, consolidation (dynamic bronchograms present) [14]. Each region was scored according to the worst pattern observed, giving a total Lung Ultrasound Score ranging from 0 to 36 (N = 0, B1 = 1, B2 = 2, C = 3). Further fluid boluses were withheld on detection of new-onset anterior B-lines, prespecified as three or more of the four anterior zones each demonstrating three or more B-lines within a single intercostal space, adapted from the FALLS protocol [15]. 'New-onset' denoted new anterior zone involvement relative to each patient's own baseline.

Sample size calculation

Sample size was calculated using MedCalc v19.0.7 (MedCalc Software, Ostend, Belgium). Based on an assumed area under the curve (AUC) of 0.70 for baseline CFTc against a null value of 0.50 - a conservative estimate derived from previously reported diagnostic performance of CFTc in shock and perioperative populations [11,16] - together with a two-sided alpha error of 5%, power of 80%, and an anticipated responder to non-responder ratio of 1:2, consistent with previously reported fluid responsiveness rates of 40-50% in critically ill populations [4,5], a sample size of 72 patients was estimated (24 responders, 48 non-responders). Allowing for 10% dropout, 80 patients were targeted.

Statistical analysis

Analysis was performed using IBM SPSS v23 (IBM Corp., Armonk, NY, USA). Normality was assessed within each group by the Shapiro-Wilk test; normally distributed variables are presented as mean ± SD with Student's t-test and non-normally distributed variables as median (IQR) with the Wilcoxon-Mann-Whitney U test, with the test indicated for each variable. Categorical variables are presented as n (%) with chi-square or Fisher's exact test. Diagnostic performance was assessed by ROC curves and the Youden index; cut-offs were derived from the present dataset, were not prespecified, and are reported as exploratory without internal validation. The percentage changes in CFTc and stroke volume from T1 to T2 were correlated by Spearman's rank correlation, one paired observation per patient. The analysis was unadjusted throughout, and secondary outcomes were exploratory and unadjusted for multiplicity. A p-value <0.05 was considered statistically significant.

## Results

Of the 80 patients enrolled, 32 were classified as responders and 48 as non-responders, with no dropouts, as summarized in the STARD flow diagram in Figure 1. The median age of the cohort was 30.5 years (IQR 24.0-46.2; range 18-65), with 50% of patients aged 18-30 years, and 41 (51.2%) were male. Mean height and weight were 1.58 ± 0.06 m and 57.59 ± 9.93 kg, with a median BMI of 23.06 kg/m² (IQR 20.65-25.82). Median Sequential Organ Failure Assessment (SOFA) and Acute Physiology and Chronic Health Evaluation (APACHE) II scores at inclusion were 7.0 (IQR 6.0-10.0) and 14.0 (IQR 11.0-18.0), respectively. The median interval from shock identification to inclusion was 23.5 minutes (IQR 15.0-38.0), and a median of 250 mL (IQR 200-400) of IV fluid was administered before inclusion. Non-responders had a significantly longer interval from shock identification to study inclusion (p = 0.031) and received more IV fluid before inclusion (p = 0.043) than responders; full baseline characteristics and their comparison between groups are shown in Table 1.

**Figure 1:**
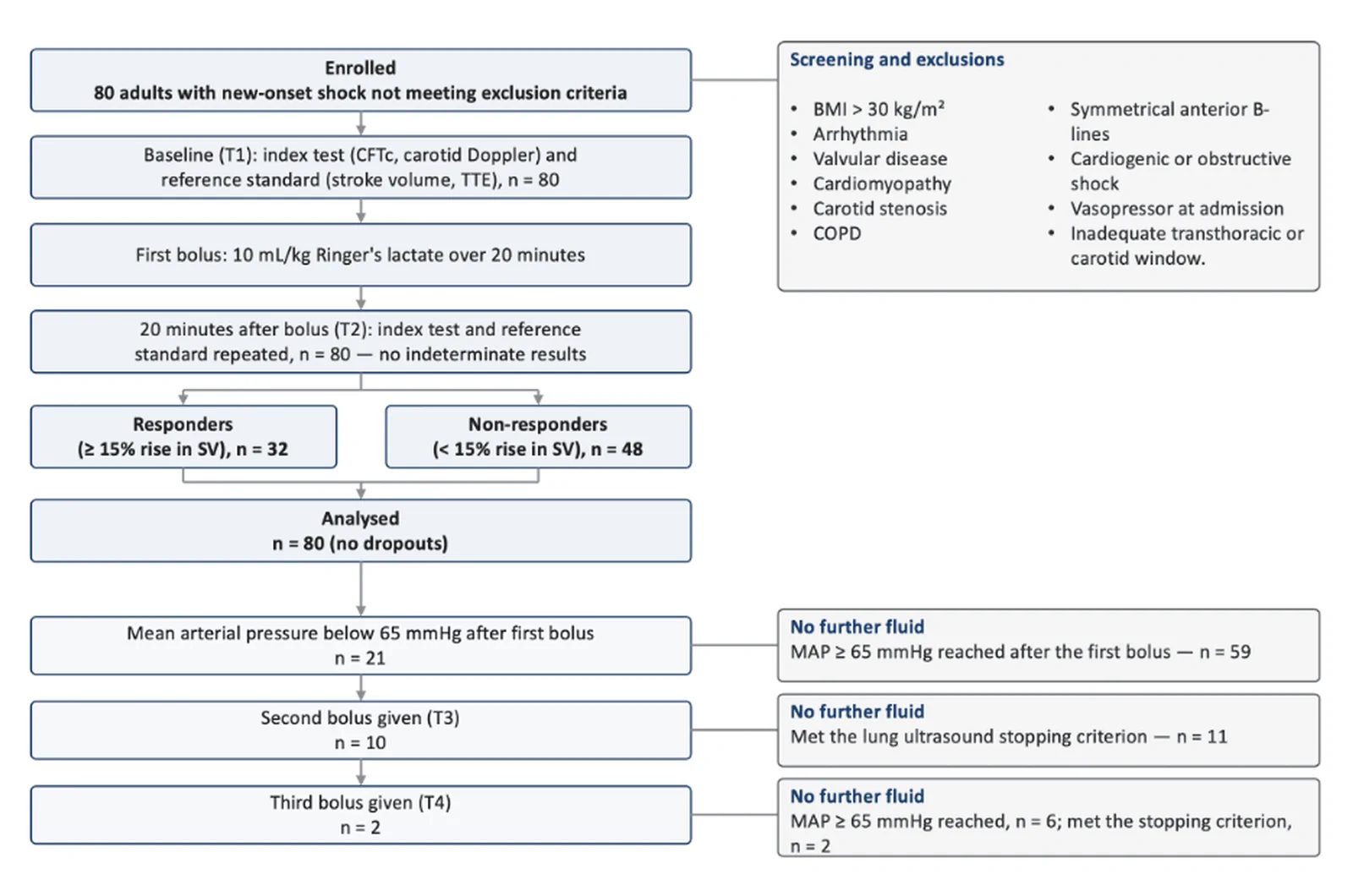
STARD flow diagram of patient enrollment, index test and reference standard acquisition, classification by stroke volume response, subsequent fluid management, and inclusion in the analysis CFTc: corrected carotid flow time; SV: stroke volume; TTE: transthoracic echocardiography; MAP: mean arterial pressure; T1: baseline; T2: 20 minutes after the first bolus.

**Table 1:** Baseline characteristics of responders and non-responders Mean ± SD, median (IQR) or n (%). Normality assessed within each group by Shapiro-Wilk; non-parametric methods used where normality failed in either group. *p < 0.05. ^a^ Wilcoxon-Mann-Whitney U; ^b^ chi-square; ^c^ Student's t-test. Focus categories are not mutually exclusive. APACHE: Acute Physiology and Chronic Health Evaluation; BMI: body mass index; CFTc: corrected carotid flow time; MAP: mean arterial pressure; SOFA: Sequential Organ Failure Assessment.

Variable	Responder (n = 32)	Non-responder (n = 48)	p
Age (years) ^a^	29.50 (20.75–54.25)	32.00 (26.00–44.25)	0.746
Male sex ^b^	11 (34.4%)	30 (62.5%)	0.014*
Height (m) ^a^	1.56 (1.53–1.58)	1.58 (1.56–1.62)	0.147
Weight (kg) ^c^	56.50 ± 10.76	58.31 ± 9.38	0.441
BMI (kg/m²) ^c^	22.65 ± 3.29	23.19 ± 3.22	0.474
APACHE-II score ^a^	13.50 (10.75–18.25)	14.00 (11.75–17.00)	0.783
SOFA score ^a^	6.50 (6.00–8.25)	8.00 (6.00–10.00)	0.136
Shock identification to inclusion (min)^a^	20.00 (15.00–30.00)	27.50 (16.75–40.00)	0.031*
Fluids before inclusion (mL) ^a^	200 (200–300)	300 (200–600)	0.043*
Probable infective focus ^b^	22 (68.8%)	39 (81.2%)	0.198
No source identified ^b^	10 (31.2%)	9 (18.8%)	—
Focus: gastrointestinal	8 (25.0%)	12 (25.0%)	—
Focus: genitourinary	4 (12.5%)	10 (20.8%)	—
Focus: respiratory	12 (37.5%)	18 (37.5%)	—
Focus: bloodstream	0 (0.0%)	3 (6.2%)	—
Focus: skin and soft tissue	5 (15.6%)	6 (12.5%)	—
Any comorbidity	18 (56.2%)	31 (64.6%)	0.454
Diabetes mellitus ^b^	4 (12.5%)	7 (14.6%)	0.791
Hypertension ^b^	6 (18.8%)	3 (6.2%)	0.083
Smoker ^b^	4 (12.5%)	6 (12.5%)	1.000
Alcohol use ^b^	0 (0.0%)	8 (16.7%)	0.015*
Malignancy / chemotherapy ^b^	4 (12.5%)	10 (20.8%)	0.337
Heart rate (bpm), T1^c^	113.81 ± 17.78	112.58 ± 22.50	0.786
Systolic BP (mmHg), T1^c^	81.47 ± 8.11	83.79 ± 7.10	0.192
Diastolic BP (mmHg), T1^a^	48.00 (46.00–50.00)	48.00 (45.75–50.00)	0.905
MAP (mmHg), T1^a^	61.00 (57.75–62.00)	60.50 (57.75–62.00)	0.805
Stroke volume (mL), T1^a^	30.79 (25.80–37.41)	38.47 (32.58–43.72)	<0.001*
CFTc (ms), T1^c^	320.99 ± 29.74	326.70 ± 36.68	0.446

A probable infective focus was identified in 61 patients (76.2%), with no source documented in 19 patients (23.8%). Overall, 49 (61.3%) patients had at least one comorbidity, most commonly malignancy in 14 (17.5%), diabetes mellitus in 11 (13.8%), and hypertension in 9 (11.2%). At inclusion, patients were tachycardic (heart rate 113.08 ± 20.63 bpm), hypotensive (median MAP 61.00 mmHg, IQR 57.75-62.00), and acidotic (median lactate 2.45 mmol/L, IQR 1.60-4.00; median base deficit −6.50 mEq/L, IQR −9.05 to −3.80). Baseline SV was significantly higher in non-responders than in responders (p <0.001). The median fluid volume received in the first 24 hours was 3390 mL (IQR 2628-3862). Vasopressors were required in 49 (61.3%) patients, initiated at a median of 75 minutes (IQR 60-100) after shock identification; noradrenaline, vasopressin, and adrenaline were used in 49 (61.3%), 18 (22%), and 1 (1.25%) patients, respectively.

For stroke volume and CFTc, all 80 patients contributed data at T1 and T2, with no indeterminate index-test or reference-standard results, but only 10 (12.5%) continued to T3 and 2 (2.5%) to T4. Stroke volume rose significantly at T2 in responders and only marginally in non-responders, whereas CFTc rose in both groups to a similar degree (Figure 2).

**Figure 2:**
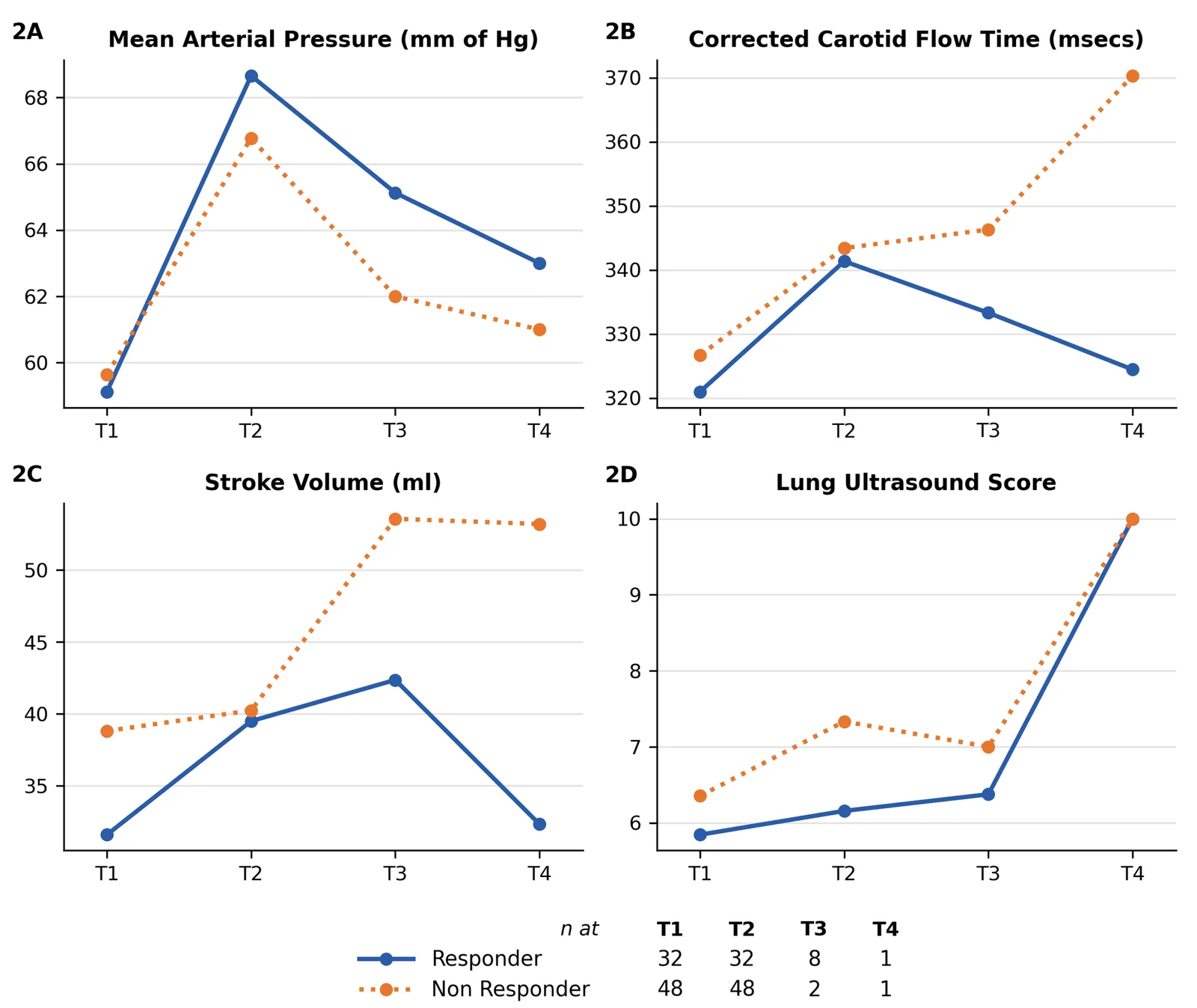
Mean arterial pressure, corrected carotid flow time, stroke volume, and lung ultrasound score in responders and non-responders across time points (T1–T4) Values are group means. T1: baseline; T2: 20 minutes after the first 10 mL/kg fluid bolus; T3 and T4: after the second and third boluses. The number of patients contributing at each time point is shown below the panels. T1 and T2 include all 80 patients. T3 and T4 include only patients who remained hypotensive without meeting a stopping criterion and therefore received further boluses (T3, n = 10; T4, n = 2). Trajectories beyond T2 are descriptive only and should not be interpreted as formal longitudinal comparisons because later measurements were available only in selected patients.

Baseline CFTc (T1) showed poor discrimination for fluid responsiveness (AUROC 0.563, 95% CI 0.435-0.691; p = 0.343). An exploratory, data-derived cut-off of ≤324.03 ms gave 71.9% sensitivity, 47.9% specificity, and an accuracy of 57.5%. The percentage change in CFTc likewise discriminated poorly (AUROC 0.568, 95% CI 0.436-0.700; p = 0.305); an exploratory cut-off of ≥7.24% gave 59.4% sensitivity, 64.6% specificity, and an accuracy of 62.5%; full diagnostic performance metrics are detailed in Table 2 and illustrated by the ROC curves in Figure 3.

**Table 2:** Diagnostic performance of baseline CFTc and the post-fluid percentage change in CFTc (n = 80) Cut-offs were derived from the present dataset by the Youden index and were not prespecified; metrics at these thresholds are optimistically biased and are reported as exploratory. p-values relate to the AUROC tested against the null value of 0.5, not to the cut-off. AUROC: area under the receiver operating characteristic curve; CFTc: corrected carotid flow time; T1: baseline; T2: 20 minutes after the first fluid bolus.

	Baseline CFTc (T1)	Δ CFTc, % change (T1–T2)
Exploratory cut-off	≤ 324.03 ms	≥ 7.24%
AUROC (95% CI)	0.563 (0.435–0.691)	0.568 (0.436–0.700)
p (AUROC versus null value of 0.5)	0.343	0.305
Sensitivity (95% CI)	71.9% (55–84)	59.4% (42–74)
Specificity (95% CI)	47.9% (34–62)	64.6% (50–77)
Positive predictive value	47.9%	52.8%
Negative predictive value	71.9%	70.5%
Positive likelihood ratio	1.38	1.68
Negative likelihood ratio	0.59	0.63
Diagnostic accuracy (95% CI)	57.5% (47–68)	62.5% (52–72)
Youden index	0.198	0.240
True positives / false positives / false negatives / true negatives	23 / 25 / 9 / 23	19 / 17 / 13 / 31

**Figure 3:**
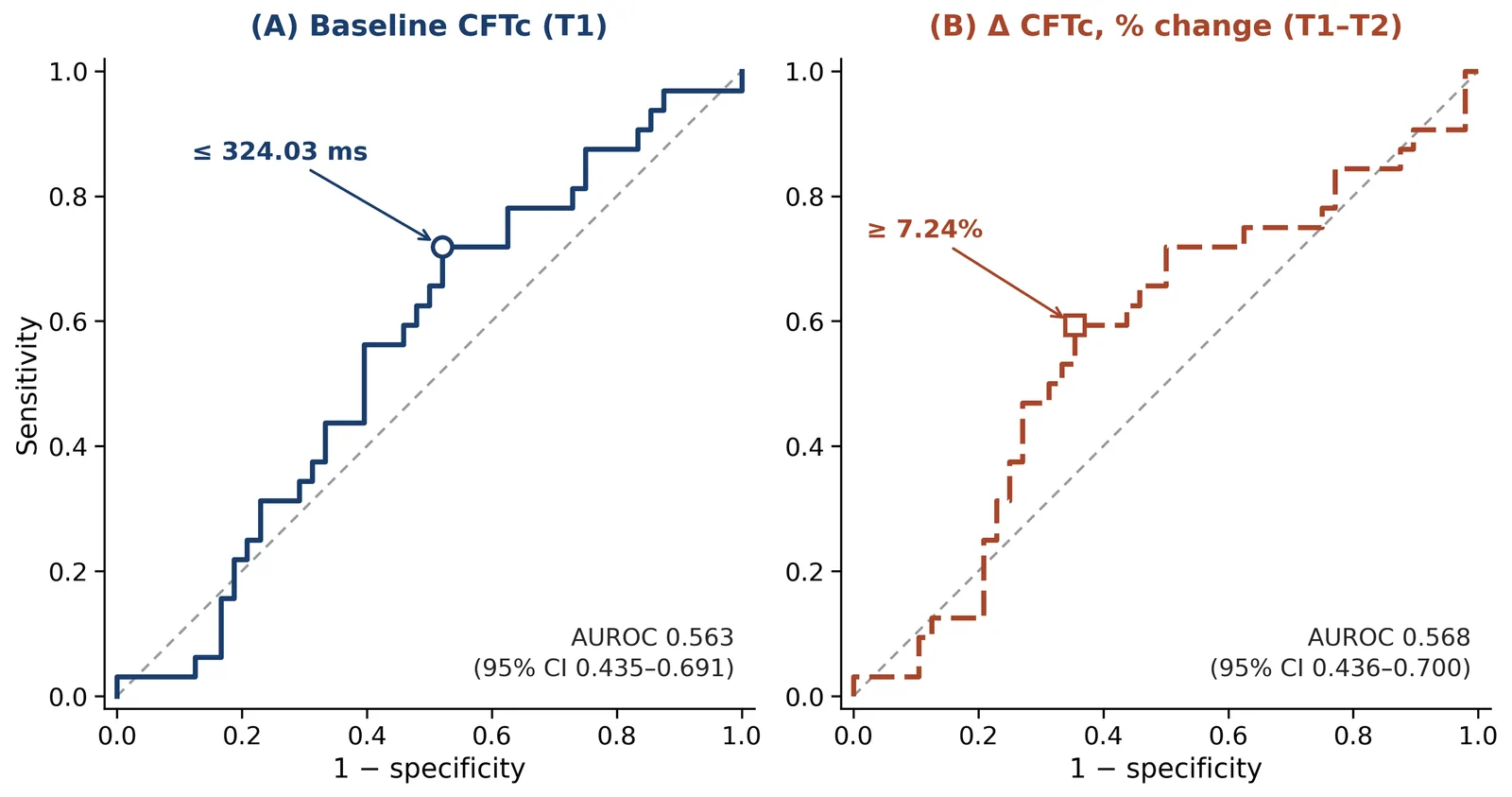
ROC curves for baseline CFTc (T1) and the percentage change in CFTc (T1–T2) in identifying fluid responders (n = 80) (A) Baseline CFTc at T1, evaluated as a predictor of fluid responsiveness. (B) Percentage change in CFTc from T1 to T2, evaluated as a concurrent post-challenge measure of the fluid response. Diagonals denote chance performance. Marked points are the exploratory, data-derived cut-offs of ≤324.03 ms and ≥7.24%; both were selected within this dataset and are not validated thresholds. AUROC: area under the receiver operating characteristic curve; CFTc: corrected carotid flow time.

Across the 80 paired data sets, the percentage change in CFTc correlated weakly and non-significantly with the percentage change in SV from T1 to T2 (Spearman rho = 0.141, 95% CI -0.081 to 0.350; p = 0.213), with each patient contributing a single paired observation; shared variance was approximately 2%. Non-responders experienced more episodes of recurrent hypotension and required earlier vasopressor initiation than responders (p = 0.031 and p = 0.025, respectively), although total fluid volume received over 24 hours was similar between groups, as summarized in Table 3.

**Table 3:** Secondary outcomes in responders and non-responders Median (IQR) or n (%). *p < 0.05. ^a^ Wilcoxon-Mann-Whitney U; ^b^ chi-square. Recurrent hypotension and vasopressor initiation identified the same 49 patients, as vasopressors were withheld until fluid responsiveness had been determined; time to vasopressor is therefore reported only among the 49 recipients. Secondary outcomes were exploratory and unadjusted for multiplicity.

Parameter	Responder (n = 32)	Non-responder (n = 48)	p
Total intravenous fluids in 24 hours (mL) ^a^	3,342 (2,642–4,325)	3,460 (2,622–3,805)	0.698
Recurrent hypotension requiring vasopressor ^b^	15 (46.9%)	34 (70.8%)	0.031*
Time to vasopressor (min), recipients only ^a^	96 (72–122)	71 (56–84)	0.025*

## Discussion

In this prospective diagnostic accuracy study, baseline CFTc showed a poor ability to predict fluid responsiveness compared with echocardiography-derived changes in SV. Baseline CFTc had limited discriminatory performance, reflected in an AUROC of 0.563 (95% CI: 0.435-0.691), and this association was not statistically significant.

Although a baseline CFTc cut-off of ≤324 ms showed moderate sensitivity (71.9%), its specificity was low (47.9%), limiting its clinical utility. These findings contrast with earlier reports from perioperative or otherwise more physiologically stable populations, where CFTc has shown considerably higher diagnostic accuracy [16,17]. Mackenzie et al. similarly found that CFT tracked volume status well in a controlled model of acute blood loss in healthy volunteers [6], while Blehar et al. and Chebl et al. reported reasonable correlations between corrected flow time and intravascular volume changes during hemodialysis or passive-leg-raise testing [7,8] - settings in which vascular tone and afterload are comparatively stable, and in which passive leg raising is a reversible maneuver rather than the actual fluid administration examined here. Our cohort, by contrast, comprised patients with predominantly early septic and/or hypovolemic circulatory failure, in whom marked and rapidly evolving derangements in vascular tone are the norm rather than the exception; differences in patient acuity, timing of assessment, and residual hemodynamic reserve may explain the weaker performance we observed.

The percentage change in CFTc after the initial fluid bolus also showed weak correspondence with the change in stroke volume. A threshold of ≥7.24% yielded modest sensitivity (59.4%) and specificity (64.6%), with an AUROC of 0.568 (95% CI: 0.436-0.700), which was not statistically significant. This contrasts with several studies reporting stronger performance for the change in carotid flow time, though most used a passive leg raise (PLR) maneuver - a reversible preload challenge distinct from the sustained fluid bolus examined here - rather than actual fluid administration. Jalil et al. reported a 24.6% rise in CFTc after PLR, with an AUROC of 0.75 [18], a discrepancy that may partly reflect their inclusion of patients already receiving vasopressors, which can augment vascular tone and sharpen the hemodynamic signal. Barjaktarevic et al., studying undifferentiated shock with non-invasive cardiac output monitoring, found that a 7 ms rise in CFTc after PLR predicted fluid responsiveness with an AUROC of 0.88 [11]. Shokoohi et al. similarly reported strong discrimination (AUROC 0.86) for percentage change in FTc after PLR in healthy, fasting adults [9]. Taken together, these more favorable results were obtained in populations or with maneuvers - PLR, vasopressor-treated shock, or healthy volunteers - that differ materially from the actively resuscitated septic and hypovolemic shock population studied here. These studies also differ in their reference standards - ranging from hydration status and controlled blood loss to non-invasive cardiac output - which complicates direct comparison of reported diagnostic performance.

An important distinction in our study is that vasopressors were initiated only after failure of fluid responsiveness by protocol-defined criteria, since vascular tone augmentation from vasoactive agents may amplify preload responsiveness and improve test performance in other cohorts [18]. Non-responders experienced recurrent hypotension more frequently and received vasopressors earlier. These exploratory findings may reflect differences in illness severity, vascular tone or hemodynamic phenotype, but they cannot establish underlying mechanism.

The relatively poor performance of CFTc in our cohort likely reflects the complex interplay between preload, afterload, and vascular tone that characterizes distributive shock. CFT is influenced not only by SV but also by arterial compliance and systemic vascular resistance, both of which are markedly altered in sepsis; this dependence on multiple, simultaneously changing variables likely limits its ability to reliably track global hemodynamic responsiveness in this population, even though it has performed better in settings - such as controlled hemorrhage or elective perioperative care - where afterload and vascular tone are comparatively stable [6-8,16,17].

Our findings are consistent with prior studies in heterogeneous emergency department and ICU populations that similarly failed to demonstrate reliable predictive value for CFTc in acutely ill patients [10]. Although a recent systematic review and meta-analysis reported that CFTc showed overall good diagnostic performance for predicting fluid responsiveness, the authors also highlighted inter-study heterogeneity and the absence of standardized cutoff values [19]. Our findings, demonstrating poor discrimination of CFTc in patients with presumed septic and/or hypovolemic circulatory failure, support the contention that the diagnostic performance of CFTc is highly context-dependent and may not be readily generalizable across different clinical populations.

This single-center study has several methodological limitations. First, a single unblinded operator enrolled a convenience sample during specific shift hours without maintaining a formal screening log. The strict eligibility criteria produced a relatively young cohort with limited cardiovascular comorbidity, introducing spectrum bias and limiting external validity to broader ICU populations. CFTc nonetheless discriminated poorly in this technically favorable population, arguing against measurement conditions alone as the explanation. Second, shock etiology was clinically presumed without formal Sepsis-3 adjudication, with a probable infective focus in 61 patients (76.2%), and follow-up was restricted to 24 hours. Because carotid flow time depends on vascular tone and arterial compliance as well as stroke volume, performance could differ between distributive and hypovolemic physiology, which were not distinguished at enrollment and could not be evaluated as subgroups. Additionally, many patients received partial fluid resuscitation prior to enrollment; unmeasured pre-hospital interventions may have confounded baseline hemodynamics and fluid responsiveness, and no adjustment was made for these differences. Third, index and reference parameters were obtained by the same unblinded clinician, and treating physicians were unblinded to clinical data. Key parameters including exact time intervals from fluid administration to post-bolus measurements were not systematically recorded, and LVOT diameter was evaluated only once at baseline. Neither intra- nor inter-observer reliability was formally quantified, though standardized acquisition protocols and three-cardiac-cycle averaging were used to minimize variation [13]. Finally, sample size considerations restricted statistical power primarily to the main ROC analysis; secondary, subgroup, and longitudinal comparisons were unpowered. The markedly reduced sample sizes at T3 and T4 reflected clinical decision-making rather than data missing at random, precluding analysis of within-patient clustering. Neither internal validation of the derived cut-offs nor sensitivity analyses were performed. Larger multicenter studies with blinded assessors and standardized criteria are needed to clarify the clinical utility of CFTc in resuscitation protocols.

## Conclusions

In this selected cohort of adults with presumed septic and/or hypovolemic circulatory failure, baseline CFTc showed poor discrimination for echocardiography-defined fluid responsiveness. The change in CFTc after fluid administration showed weak correspondence with the change in stroke volume and did not support its use as a standalone post-challenge measure. These findings should be interpreted considering the single-center design, restricted eligibility criteria, prior fluid exposure, and absence of formal measurement-reliability assessment.
